# 

**Published:** 1852-06

**Authors:** 


Philadelphia.

### CONTENTS

OF THE

### MEDICAL EXAMINER.

NEW SERIES—NO. XC.—JUNE, 1852.

### ORIGINAL COMMUNICATIONS.

Taliacotian Operation. By Joseph Pancoast, M. D., Prof. of Anatomy in Jefferson Medical College. Reported by Charles P. Turner, M. D., of Philadelphia, - - - - 341

Influence of the Imagination of the Mother upon the Fœtus. By L. Slusser, M. D., of Canal Fulton, Ohio, - - - 344

Case of Poisoning by Oleum Gaultheriæ. By Thomas J. Gallaher, M. D., of Pittsburgh, Pa., - - - - - - 347

Mortality of Philadelphia for January, February and March, arranged from the Record kept at the Health Office. By Wilson Jewell, M. D., - - - - - - - 350

Notes on Bedford Springs, and the Diseases in which a resort to them is useful. By Caspar Morris, M. D., of Philadelphia, - 358
Modification of the Bivalve Speculum. By Joseph Smith, M. D., of Danville, Ky., - - - - - - - 367
The Professional Education of Dentists. By E. B. Gardette, M. D., Dentist, - - - - - - - - 368
On the Reparative Power of the Spinal Cord after complete division. By E. Brown-Séquard, M. D., of Paris, - - - 379

### CLINICAL REPORTS.

Clinic of the Jefferson Medical College. Service of Professor Dunglison. Reported by J. Aitken Meigs, M. D., - - 383

### EDITORIAL.

Editorial Courtesy, - - - - - - - 391
Dr. Brown-Séquard's Lectures, - - - - - 391
Appointment of Dr. John Neill, - - - - - 391

### MEDICAL NEWS.

American Medical Association: Abstract of Proceedings of its late Meeting held at Richmond, Va., May 4, 1852, - - 392
Proceedings of the Medical Society of the State of Pennsylvania at its Annual Session, Philadelphia, May, 1852, - - 398
The Voltaic Pile, - - - - - - - 412
Number of Physicians in Paris, - - - - - 412
Novel Treatment of Aneurism, - - - - - 412

